# The computational power of the human brain

**DOI:** 10.3389/fncel.2023.1220030

**Published:** 2023-08-07

**Authors:** Peter J. Gebicke-Haerter

**Affiliations:** Institute of Psychopharmacology, Central Institute of Mental Health, Faculty of Medicine, University of Heidelberg, Mannheim, Germany

**Keywords:** artificial and biological intelligence, analog-digital computation, cellular computation, molecular computation, network oscillations, learning and memory, engrams, bifurcations

## Abstract

At the end of the 20th century, analog systems in computer science have been widely replaced by digital systems due to their higher computing power. Nevertheless, the question keeps being intriguing until now: is the brain analog or digital? Initially, the latter has been favored, considering it as a Turing machine that works like a digital computer. However, more recently, digital and analog processes have been combined to implant human behavior in robots, endowing them with artificial intelligence (AI). Therefore, we think it is timely to compare mathematical models with the biology of computation in the brain. To this end, digital and analog processes clearly identified in cellular and molecular interactions in the Central Nervous System are highlighted. But above that, we try to pinpoint reasons distinguishing *in silico* computation from salient features of biological computation. First, genuinely analog information processing has been observed in electrical synapses and through gap junctions, the latter both in neurons and astrocytes. Apparently opposed to that, neuronal action potentials (APs) or spikes represent clearly digital events, like the yes/no or 1/0 of a Turing machine. However, spikes are rarely uniform, but can vary in amplitude and widths, which has significant, differential effects on transmitter release at the presynaptic terminal, where notwithstanding the quantal (vesicular) release itself is digital. Conversely, at the dendritic site of the postsynaptic neuron, there are numerous analog events of computation. Moreover, synaptic transmission of information is not only neuronal, but heavily influenced by astrocytes tightly ensheathing the majority of synapses in brain (tripartite synapse). At least at this point, LTP and LTD modifying synaptic plasticity and believed to induce short and long-term memory processes including consolidation (equivalent to RAM and ROM in electronic devices) have to be discussed. The present knowledge of how the brain stores and retrieves memories includes a variety of options (e.g., neuronal network oscillations, engram cells, astrocytic syncytium). Also epigenetic features play crucial roles in memory formation and its consolidation, which necessarily guides to molecular events like gene transcription and translation. In conclusion, brain computation is not only digital or analog, or a combination of both, but encompasses features in parallel, and of higher orders of complexity.

## 1. Information processing in brain: theoretical concepts

The brain has always been compared with a highly sophisticated computer. To this end, scientists and computer technologists have been working jointly and in parallel to unravel structural and functional connectivities and dynamics of communication and information processing in the Central Nervous System. Toward the end of the last century, computer technology began to focus almost exclusively on digital information processing. And, indeed, many events in the CNS are running in all-or-none, or digital manners, as well.

### 1.1. Early concepts: turing machine and reservoir computing

Despite different firing rates, all-or-nothing action potentials or spikes could be used for applications of mathematical algorithms in artificial neural networks (ANN) including series of discrete instructions based on Turing’s work Turing (1936). In his mathematical analysis of algorithms, Turing assumed discrete time-steps and discrete variables for computation [Turing-machine (TM)]. Consequently, the question has been raised, if the brain can be compared to a TM. However, in contrast to the algorithmic system of a TM, very often the human mind is facing the problem to *prove the truth of propositions*. Its solution necessarily includes procedures that take into account their *meaning*, e.g., not just reading a text, but reading “between the lines.” Those procedures defined as *semantical*, can be activated in the human brain. This process enables the brain to *prove* the notion of “meaning” (as condition of truth). In other words, the human mind can associate the notion of prove with that of meaning, which contrasts with a TM. This assertion, however, has been vividly disputed and rejected [e.g., Kerber (2005)].

Analog computation, hence, contrasts profoundly with algorithms implemented in a TM. The great power of analog computation was also appreciated later by Von Neumann (1958) and Turing (1990), who investigated analog computation in brains and in cells, respectively. Additional work highlighting analog computation in the CNS was published at the same time (Tank and Hopfield, 1987). However, both analog and digital computing may be reconciled by analog-digital crossover. The fundamental reason for a substantial improvement of performance through analog–digital crossover lies in information theory: in the digital approach, information is encoded by many 1-bit interacting computational channels but in the analog approach by only one multi-bit computational channel (Sarpeshkar, 1998). In the end, the digital approach distinguished by high informational precision cannot compete with the lower informational precision in analog computation where all the bits are processed in parallel and the task is solved right away.

From that it may be concluded that the human CNS has developed ways of computation that cannot be reduced to the workings of a TM (Toni et al., 2007), because complex brain activities, like abstraction and mentation, require more “elastic” forms of computation (Arbib, 1987) far above any of today’s machine learning techniques. More sophisticated information processing is needed such as hybrid computation, joining discrete and continuous forms of communication.

It is essential for the brain to create appropriate behavior based on relatively small amounts of information. To this end, it is making use of unsupervised learning as opposed to supervised learning. In the latter, the system is supplied with the correct answers to model, whereas in the former the learning system finds structural patterns on its own without guidance, i.e., there is no “training set” to learn from, or in other words, to find statistically “independent” components within the input signal.

In fact, the CNS permanently has to analyze complex events in a steadily changing environment, where incoming stimuli are lacking any preset “label” or category (Popper and Eccles, 1977; Edelman, 1987). It has been proposed that those environmental signals have to be categorized by computational maps as intermediate steps of information processing (Knudsen et al., 1987). In such computational maps, a systematic variation in the value of the incoming physiological parameters occurs across at least one linear dimension of the neural structure. Groups of neurons belonging to a map can be viewed as analytical processors, filtering incoming signals in slightly different ways dependent on cellular responsiveness to the stimulus and operating jointly and in parallel. In that manner, the environmental input is converted into a place-coded, probability distribution of cellular activation states. This parallel information processing has been put forward as a basic requirement for global map formation in Gerald Edelman’s, Extended Theory of Neuronal Group Selection (Edelman, 1989). On those grounds, it has been hypothesized that representations of complex memories are distributed and stored throughout the brain (Lashley, 1950; Hübener and Bonhoeffer, 2010; Josselyn et al., 2015), although the mechanisms of their formation are still enigmatic.

The vertebrate CNS contains a number of anatomical structures functioning not only as negative but also as positive feedback systems. For instance, the hypothalamus continuously releases neural and humoral signals processed within a black box of the target cells. This may result in either lowering (negative feedback) or enhancing (positive feedback) the discrete (neural) output. Those feedback systems are intrinsically connected by recurrent 3-dimensional neural networks that may or may not require any equivalent of full backpropagation through a multilayer network. Within a computer environment, back propagation algorithms have been implemented to detect and correct input layer errors in multi-layer neural networks, e.g., in reservoir computing (RC). As basis sets (or “reservoirs”), randomly connected recurrent networks, like “liquid-” (Maass et al., 2002) or “echo-state machines” (Jaeger and Haas, 2004) have been constructed. A delay-based mixed analog and digital implementation of RC with a non-linear analog electronic circuit as a main computational unit meets the requirements of high dimensionality, which lies in the many degrees of freedom introduced by the delay time τ (Lakshmanan and Senthilkumar, 2011). Although the reservoir itself (the non-linear delay system) is analog, the input and readout are still digital. Reservoirs of random non-linear filters are one approach to close in to the various tuning properties of many neurons, encompassing high dimensionality and mixed selectivity, as observed in the prefrontal cortex (Enel et al., 2016). The leading hypothesis is that storage of memories is reflected in the connection strengths between neurons (Crick, 1984), and learning and storing new memories modify these strengths (Hebb, 2005). An elegant model of memory devised in the computer is the Hopfield network (Chaudhuri and Fiete, 2016). Learning in a Hopfield network (Hopfield, 1982, 1984) is like presenting a new memory network to a noisy version of a previously stored fundamental memory. Comparing those networks, new attractors in the configuration space of the system equivalent to non-linear adaptation to the best fit are constructed. When the configurations of the systems are sufficiently close, they dynamically relaxe toward the nearest fundamental memory, and stay there indefinitely. But simulations of neuronal interactions in the brain, constructing artificial neuronal networks (ANN) and introducing supervised and unsupervised learning algorithms resulting in systems of artificial intelligence (AI) still left many questions unanswered.

### 1.2. Artificial intelligence

At this point, it is timely to evaluate the basic principles of AI, where it stands presently, and to compare it with the biological facts known until now about information processing and storage (memory) in the CNS.

Let’s start with “Moravec’s paradox” (Moravec, 1988), that states: “It is comparatively easy to make computers exhibit adult level performance on intelligence tests or playing checkers, but difficult or impossible to give them the skills of a 1-year-old when it comes to perception and mobility.” “The main lesson of more than thirty-five years of AI research is that the hard problems are easy and the easy problems are hard.”

But the fundamental idea that neurons stand out with a capacity of analog computation, similar to adaptive non-linear processing units (McCulloch and Pitts, 1943), is not well covered by the toolbox of formal logic (Rosenblatt, 1957). The next generation of intelligent systems has to be endowed with sources for good implicit biases able to make smart generalizations across varying data distributions and be able to learn new tasks quickly without forgetting previous ones.

In contrast to biological brains, only neurons are considered in ANNs (Titley et al., 2017). Moreover, they clearly lack some crucial generalization capabilities. One of those is a lack of robustness of the networks to “minimal adversarial perturbations” even when using the simplest toy datasets of machine learning, such as MNIST (Szegedy et al., 2013). Apparently, the details of network structure at both a coarse (e.g., connectivity between hidden layers) and a fine scale (e.g., cell types, non-linearities, or even dendritic computation and ion channel functions) are at present insufficiently represented according to the available neuroscience data (Markram, 2006).

Nevertheless, construction of ANN included properties of biological networks, such as normalization, winner-takes-all mechanisms like max pooling (Riesenhuber and Poggio, 1999), attention (Larochelle and Hinton, 2010), dropout (Srivastava et al., 2014), or simply implemented neurons as basic computational elements. However, there are many important features lacking in ANN: for example, an artificial neuron in the machine learning literature is considered as a point neuron. Neuronal spikes, or action potentials have been considered as the minimal units of information generated by a neuron. Analogous to bits in computers, the spike was associated with an “all-or-none” digital phenomenon. Neurons as nodes in ANN were assigned with discrete, repetitive electrical spikes as inputs and emission of electric signals at the output site. Each cycle of their activation obeyed a sigmoidal function whereas activation of biological neurons is more graded depending of the incoming stimuli over time. Information flow in ANN is only unidirectional from input to output. In analogy to digital units they produce an action potential, or not. There is no graded action potential. Or, as depicted by Von Neumann (1951), “The nervous pulses can clearly be viewed as two-valued markers, characterized by the binary digits 0 and 1.” There are, indeed, some events in neuronal communication showing very stable action potentials (Sierksma and Borst, 2017). But for most neuronal cell types, these two assertions are incorrect. For example, spike frequencies have to be taken into consideration. One presynaptic neuron may discharge repetitive, monotonous spikes, another may encrypt its firing rates reminiscent of the MORSE-alphabet (Borst and Theunissen, 1999). Hence, each neuron may have its special firing rates (language) distinct from others, dependent on environmental impact (spike timing: Gütig, 2014). Fine homeostatic adjustments of membrane voltage may impact on the generation of action potentials which may not qualify as computation (Stuart et al., 1997), but encode the “symbols,” or the “alphabet” used by the brain to compute. Therefore, more recently spiking neural networks (SNN) have gained more interest due to their closer similarities to biological neural networks and to their lower energy consumption. They can be used to attain advanced cognitive capabilities when basic mechanisms of synaptic plasticity are implemented by neuromorphic engineering, e.g., by using IBM’s TrueNorth neuromorphic hardware (Walter et al., 2015). Their computational power surpasses the abilities of ANN in that they can process spike trains over time decoding temporal information. Moreover, implementation of SNNs even on large scales is not difficult (Cessac et al., 2010; Pietrzak et al., 2023).

Various numbers of inputs (edges) are associated with various weights and their weighted sum or activation is transformed into a scalar non-linear function (ReLU, ELU, sigmoid, etc.) to produce the (yes/no) output. Inputs are external signals and outputs may recognize those signals. Nevertheless, owing to the remarkable increase of capacities of electronic devices and development of new technologies such as 3D integrated circuits, nano-scale transistors, memristors, or phase-change materials and organic electronics, AI has entered a more sophisticated level, taking into account more biological features, with the promising approach of neuromorphic engineering (Indiveri and Horiuchi, 2011; Brivio et al., 2019; Yang et al., 2020; Gandolfi et al., 2022). Simulations showed encouraging results where a cerebellum-inspired neuromorphic architecture was mapped into a large-scale cerebellar network to explore cerebellar learning (Yang et al., 2022). Moreover, canonical neural networks (CNN) have been constructed apparently reducing the cost function and minimizing variational free energy by modulating synaptic plasticity with some delay (Isomura et al., 2022; Fields et al., 2023).

Despite those advancements, energy consumption in high-dimensional, multi-layer ANNs or SNNs is extremely high compared to biological networks. In contrast to biological learning, which is local, machine learning impacts on all elements of ANNs. Machine learning has been implemented in practically all AI applications (Kassanos, 2020). Parameters of a flexible non-linear function are adapted to optimize an objective (goal) that depends on data. This optimization is usually implemented, e.g., in ANN, by backpropagation, an algorithm developed by Paul Werbos in his Ph.D thesis in Werbos (1974). Backpropagation is a fast algorithm of learning, displaying changes of the cost function in a network, when changing any weight of inputs (Rumelhart et al., 1985). It is used very often for learning in recurrent neural networks (RNN), where data from time series have to be retained to be used for subsequent steps.

For example: a simple optimizing procedure of a network’s performance is to apply the “twiddle” algorithm or, more technically, “serial perturbation.” This means that a single weight is perturbed (i.e., “twiddled”) with a small increment, and improvement is noted if the cost function has improved compared to the weight unperturbed. In terms of modeling, negative feedback signals require: (a) an input of quantity K from an external source, fed into the black box of the system with a circuitry S, that connects the source to a target, (b) the target, that steadily feeds back its output value of K’, whose value is close to that of K, to the circuitry S. An error detector implanted in S calculates the error signal E = K–K’. E then is able to adjust the entire system along with improvement of its performance. The ultimate adjustment of the system is reached when K and K’ are equal and E is zero (Wiener, 1961). The computational power of S probably relies on continuous rather than discrete values.

Apart from the details outlined above, some important distinctions between ANNs vs. biological networks have to be highlighted: processing time is faster in ANNs, there is no refractory period, but processing is serial not parallel, network architecture is determined by the designer, ambiguity of incoming data is not tolerated (fault intolerant), activation obeys sigmoidal functions whereas activation of biological neurons is slower and better tuned to strength of input, energy consumption is orders of magnitude higher in ANN to solve similar tasks (brain approx. 20 watts vs. 250 watts only for running a GeForce Titan X GPU), and they produce a lot of heat during computation (50–80 vs. 36.5–37.5 degrees Celsius), ANN are composed of a few hundreds to a few thousands of neurons in contrast to approx. 86 billions of neurons and 100 trillions of synapses in biological networks, physical units are transistors and not neurons, and all functions including learning are not autonomous but have to be programmed.

After more than 60 years of AI research, Moravec’s paradox has not been solved.

Real neurons are more sophisticated machines. Moreover, cerebral microcircuits may encompass various types of neurons that are genetically and functionally distinct (Douglas and Martin, 1991; Jiang et al., 2015). Each one may perform operations like gating, homeostatic regulation, and divisive normalization.

Our brain can easily perform tasks like grasping, navigation, and scene understanding, which are tasks of subconscious intelligence hard to teach to machines (Sinz et al., 2019). The brain’s adaptive capacity persists into adulthood, and entails higher-order cognitive functions, such as learning and the formation of memories (Weinberger, 1995; Sanes and Donoghue, 2000; Chklovskii et al., 2004; Pinaud et al., 2005; Yao and Dan, 2005). Understanding how sensory experience affects the functional organization of the vertebrate brain requires deep insights into ways of activation of neuronal ensembles and more knowledge about influences of experiential factors on neurochemically distinct cell types. Additionally, the development of coordinated gene expression programs that establish stable, long-term changes in neuronal performance have to be considered.

## 2. Information processing in brain: biological concepts

### 2.1. Electrical synapses and neuronal gap junctions as fundamentally analog devices

At this point, we want to proceed from theoretical *in silico* concepts to potential capacities of cellular and molecular structures of the CNS, outlining similarities and differences to achievements made with electronic devices. Synaptic processes have been considered as key events in information processing and storage in the brain. They can be divided into vesicular release-dependent and direct electrical transmission systems. The existence of the latter has been a matter of debate for a long time, because neuronal gap junctions in mammalian CNS were hard to identify by thin-section electron microscopy (EM). When, later on, those gap junctions were found (Rash et al., 1996; Kamasawa et al., 2006), their small sizes did not conform with prevailing ideas to serve for rapid and efficient intercellular propagation of action potentials (Dewey and Barr, 1962, 1964; Loewenstein, 1966, 1981). More evidence confirmed existence of electrical synapses during early stages of mammalian brain development, such as in neo-cortex (Peinado et al., 1993a), retina (Penn et al., 1994), and spinal cord (Walton and Navarrete, 1991). Those connections were considered to establish functional compartments and early neuronal networks (Yuste et al., 1992; Kandler and Katz, 1998), but would disappear in the course of brain and spinal cord development (Peinado et al., 1993b). However, those types of synapses have also been identified in many areas of adult brain, where they may function as low pass filters (Connors and Long, 2004). The gap junction channel proteins Cx36 and Cx45 were detected in ultrastructurally defined gap junctions in retinal and spinal cord neurons (Rash et al., 2000, 2001a,b; Li et al., 2008). Additionally, mRNA expression for the connexins Cx45 and Cx57 was reported from various neurons (Hombach et al., 2004; Maxeiner et al., 2005; Schubert et al., 2005; Dedek et al., 2006; Van Der Giessen et al., 2006; Ciolofan et al., 2007; Palacios-Prado et al., 2009). Hence, gap junctions, fulfilling analog information transduction, that abundantly occur between mammalian neurons (Kamasawa et al., 2006; Rash et al., 2007a,b), may also execute as-yet-undetermined electrical, ionic, or metabolic functions (Gilula et al., 1972) other than propagation of action potentials. Resistance and time constants of the coupled cells as well as the conductance of the gap junction control the strength of electrical transmission (Bennett, 1966). That means, that the time constant of a postsynaptic cell can attenuate high frequency-containing signals such as spikes, but may have low impact on longer lasting, low frequency-containing signals.

Typically, transmission at electrical synapses is bidirectional, which results in spreading of changes of cellular membrane potentials to all the partners within an electrically-coupled compartment (Wheal and Thomson, 1984), which is reminiscent of computer models of ANNs. This also includes subthreshold responses, such as synaptic potentials (Zsiros et al., 2007) as well as spontaneous oscillations (Placantonakis et al., 2006). It has been put forward that “brain oscillations are generated in almost every part of the brain,” and that “network oscillations may assist to store and retrieve information in synapses and regulate the flow of information in neural circuits” (Gelperin, 2006; Kahana, 2006; Paulsen and Sejnowski, 2006; Sejnowski and Paulsen, 2006). In this way, electrical synapses are considered to be pivotal for information processing, learning and memory, and human consciousness in the CNS (Nagy et al., 2018), displaying mechanisms of computations that are fundamentally analog.

In hippocampal pyramidal cells, electrical synapses between inhibitory interneurons facilitate synchronous high-frequency γ-oscillations. In GABAergic interneurons in striatum (Fukuda, 2009) and cortex (Fukuda, 2007), electrical coupling has been shown to synchronize activity in interneuronal networks and in neocortical pyramidal cells (Diesmann et al., 1999; Galarreta and Hestrin, 1999; Gibson et al., 1999; Deans et al., 2001; Blatow et al., 2003; Hestrin and Galarreta, 2005; Fukuda et al., 2006). Fast spiking basket cells (FS BCs) are one of the major types of hippocampal and neocortical interneurons (Freund and Katona, 2007; Klausberger and Somogyi, 2008; Hu et al., 2010). There is increasing evidence that FS BCs are important in controlling executive functions, such as working memory and attention, and also play a role in neurodegenerative disorders (Baeg et al., 2001; Kann, 2016; Kim et al., 2016). However, a number of studies concluded that FS BCs serve as “on–off” cells (Chiovini et al., 2014) that integrate inputs in linear–or at best sublinear ways - like point neurons (Martina and Jonas, 1997; Hu et al., 2014). This point of view completely ignored potential dendritic influence. Therefore, FS BCs, similar to pyramidal neurons (Poirazi et al., 2003a), can be better envisaged by a two-stage integrator abstraction than as a point neuron. Identification of neuronal gap junctions in excitatory glutamatergic cortical and hippocampal pyramidal cells has been taken as evidence for abundant electrical synapses in those cells (Mercer et al., 2006; Wang et al., 2010). Likewise, this type of synapses has been found in noradrenergic locus coeruleus neurons (Travagli et al., 1995), and between inhibitory interneurons (Kosaka, 1983; Fukuda and Kosaka, 2000a,b). In the suprachiasmatic nucleus Cx36-containing neuronal gap junctions (Rash et al., 2007a,b) are required for normal circadian behavior, and loss of these gap junctions (in Cx36 null mice) affects circadian rhythms (Jiang et al., 1997; Long et al., 2005). In hypothalamus, electrical synapses between magnocellular neurons are involved in pulsatile oxytocin release by synchronizing burst firing (Hatton et al., 1988; Yang and Hatton, 1988; Hatton, 1997; Hatton and Zhao Yang, 2002).

### 2.2. Spike shapes and synaptic transmission

When spikes arrive at the presynaptic terminal, they provoke the opening of voltage gated calcium channels (Cav), with subsequent increase of intracellular Ca2 + concentration and vesicular neurotransmitter release into the synaptic cleft, which are quantal, digital events (Katz, 1969). The shape and time course of the AP depolarizing the nerve terminal membrane modify the gating of calcium channels and the magnitude of calcium flux (Klein and Kandel, 1980; Llinas et al., 1981; Spencer et al., 1989; Augustine et al., 1991; Pattillo et al., 1999). Already small variations in presynaptic calcium release may significantly impact on strength of synaptic transmission, because of the power law relationship between intra-terminal Ca2 + concentration and neurotransmitter release (Sabatini and Regehr, 1997; Bollmann et al., 2000; Bischofberger et al., 2002; Fedchyshyn and Wang, 2005; Yang and Wang, 2006; Bucurenciu et al., 2008; Scott et al., 2008; Neishabouri and Faisal, 2014). Those subtle variations of incoming action potentials do not obey all-or-nothing rules, hence are analog reactions. Further aspects are covered below in (“3. The postsynaptic element and dendritic computation”).

All of them serve to accumulate voltage in the postsynaptic neuron, which triggers discharge of an action potential when a critical threshold, specific for each neuron, is overcome.

Incoming action potentials may vary both in amplitude and width adding to complex signals in neuronal computation. They are both digital and analog entities. First, reduced spike amplitudes typically result from decline of conductance of voltage-gated sodium channels (Nav), which may be due to repetitive firing, as observed in long term potentiation (LTP) (Brody and Yue, 2000; Prakriya and Mennerick, 2000; Ma et al., 2017; Ohura and Kamiya, 2018). Reduced spike amplitudes diminish synaptic transmission as shown at hippocampal (He et al., 2002) and cerebellar synapses (Kawaguchi and Sakaba, 2015).

**FIGURE 1:**
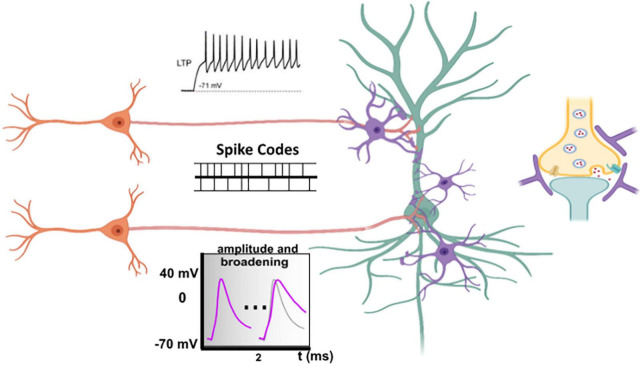
Long-term potentiation, spike codes and spike broadening. Opening times of calcium channels and the magnitude of the calcium flux in the presynaptic membrane not only depend on the time course (spike codes) but also on the shape of the incoming action potential (AP) (Llinas et al., 1981; Augustine et al., 1991; Pattillo et al., 1999). Subtle changes in calcium influx characteristics fine-tuned by both spike codes and shape of APs can precisely proportionate transmitter release. The speed and magnitude of calcium entry in the presynaptic terminal during an AP is highly dependent on the time of repolarization. Voltage-gated potassium channels are responsible for repolarization. Impairment those channels results in AP (Spike) broadening, subsequent increased calcium influx, and transmitter release. Long-term potentiation (LTP), which is associated with repetitive firing, may not only suppress conductance of voltage-gated potassium channels (Kv), but also of voltage-gated sodium channels (Nav), which typically results in reduced spike amplitudes. Altogether, one can conclude that incoming APs at the presynaptic terminal may be stereotypic, discrete signals, but can also be graded inputs more equivalent to analog information.

Second, the speed and magnitude of calcium entry in the presynaptic terminal during an AP is highly dependent on the time course of the repolarization phase, which is under control of potassium release. Therefore, AP broadening with subsequent enhanced calcium influx and transmitter release has been observed upon blockade of voltage-gated potassium channels (Figure 1; Augustine, 1990; Wheeler et al., 1996; Shao et al., 1999; Faber and Sah, 2003; Kim et al., 2005; Liu et al., 2017). For example, spike broadening during repetitive firing results in reinforcement of synaptic transmission in the pituitary nerve (Jackson et al., 1991), in dorsal root ganglion (Park and Dunlap, 1998), and in mossy fibers (Geiger and Jonas, 2000). Moreover, neuromodulators, like glutamate and GABA may lower Kv channel conductances in hippocampal neurons, eliciting increased synaptic transmission by depolarizing axonal membrane potential and spike broadening (Ruiz et al., 2010; Sasaki et al., 2011).

Thirdly, AP broadening is also influenced by the density of voltage-gated channels, which may be heterogeneous along the axon. This has been shown in cerebellar stellate cell interneurons for peri-terminal Kv3 channels (Rowan et al., 2016).

Furthermore, dopamine D1 receptor activation may induce decrease in Kv1-dependent ID current and spike broadening in cortical pyramidal neurons upon (Dong and White, 2003; Yang et al., 2013). Those admittedly small effects on shapes of neural spikes are completely different from what we find in digital computers. The phenomenon has been called “analog-digital synaptic transmission” (Clark and Häusser, 2006; Alle and Geiger, 2008; Debanne et al., 2013; Rama et al., 2015; Zbili et al., 2016). Consequently, APs cannot be considered as purely digital events.

Needless to mention that spike broadening and subsequent increased synaptic release due to Kv channel down-regulation has been identified in various neurologic disorders such as schizophrenia, episodic ataxia type1, fragile X syndrome, autism, and epilepsy (Deng P. Y. et al., 2013; Begum et al., 2016; Crabtree et al., 2017; Vivekananda et al., 2017; Scott et al., 2019).

## 3. The postsynaptic element and dendritic computation

As described above, learning occurs by implementing optimization algorithms, comparing a prediction with a target, and the prediction error is used to drive top-down changes in bottom-up activity. In contrast to circuit-level computations that use interactions between point-like neurons with single, somatic non-linearities (Gómez González et al., 2011), more advanced studies have taken into account complex and non-linear capabilities of information processing within the dendritic tree of cortical neurons (dendritic computation) (for overview see: Cuntz et al., 2014). Stimulation of multiple synapses in a single dendrite may result in variations of supralinearity of electrical integration and amplitudes of EPSPs depending on synapse location. In contrast to the base or the middle section of the dendrite, the tip displays higher gain, higher EPSP amplitude, and higher EPSP supralinearity (Branco and Häusser, 2011). Moreover, the positioning of excitation along the dendrite affects the amplitude and threshold of basal dendritic spikes (Behabadi et al., 2012). Proximal excitation enhances the voltage gain but diminishes the threshold of distal inputs, whereas in more proximal inputs distal excitation lowers the threshold for dendritic spike generation. Hence, modulation of dendritic excitability along with changes in the spatial wiring of synaptic connections may be viewed as optional ways to store memory in the brain (Chklovskii et al., 2004). Three main types of dendritic spikes can be distinguished: sodium, calcium and NMDA (N-methyl-D-aspartate) spikes. There is ample evidence of their occurrence in pyramidal neurons.

In addition to dendritic spiking events, more analog forms of communication have to be mentioned, such as the influence of subthreshold potentials on effects of action potentials (Clark and Häusser, 2006), transmission of voltage signals through gap junctions (Vervaeke et al., 2012), or ephaptic coupling between neighboring cells (Anastassiou et al., 2011). These may be due to slow membrane potential dynamics, to close proximity of interacting cells, or to large degrees of population synchrony (Sengupta et al., 2014). This led to the “2-layer” model of neuronal integration. First, terminal dendrites represent non-linear and independent thresholding units. Then, the combined output has to pass a second threshold at the cell body (Poirazi et al., 2003b). Hence, the postsynaptic neuron is a multi-task element within the neuronal network that may receive more than thousand messages from other neurons both on its dendrites and cell body (Figure 1). However, in contrast to earlier views that the cell body makes the decisions, which are digital, it turned out later that dendrites are responsible more often in decision-making than the cell body (London and Häusser, 2005). Those computations are both digital and analog. In terms of non-linear inhibitory and excitatory inputs in active dendrites, it has been shown that their excitability is under powerful control of local inhibition (Gidon and Segev, 2012; Jadi et al., 2012; Lovett-Barron et al., 2012; Müller et al., 2012; Wilson et al., 2012). Local clustering of synaptic connections in dendritic branches, however, may impact significantly on synaptic modifications (Branco and Häusser, 2010). This clustered synaptic plasticity has been associated with increased storage capacity and feature binding (Poirazi and Mel, 2001; Govindarajan et al., 2006; Legenstein and Maass, 2011). The arrangement of synapses in clusters likely stabilizes long-term memories, because clustered spines were more stable than isolated ones. If presynaptic neurons become correlated, the optimal response becomes non-linear. Non-linear dendrites are essential in neural network computations with their capacities to decode complex spatio-temporal spike patterns. Thus, inputs from presynaptic neurons with correlated activities are integrated non-linearly, while inputs from uncorrelated neuronal activities are integrated linearly (Larkum and Nevian, 2008). This is achieved in the same dendritic tree by clustered synapses of correlated inputs (Harvey and Svoboda, 2007). In other words, there is non-linear summation of synchronous, adjacent inputs on the same dendritic branch, whereas more remote and separated inputs undergo linear combination. Consequently, presynaptic neurons with strongly correlated activities are in contact with nearby locations on dendrites whereas independent neurons are connected to distinct dendritic subunits. The optimal response can be expressed as a set of non-linear differential equations that requires storing and continuously updating ∼N2 variables within the dendritic tree, where N is the number of synapses.

Moreover, repetitive presynaptic inputs typically reduce responses, whereas APs dissimilar to the recent spiking history cause larger changes. Additionally, changing spike frequencies, e.g., highly synchronized spikes superimposed on few, randomly occurring spikes (quiescent states) can evoke supralinear integration (Gasparini and Magee, 2006).

In this view, synaptic clusters from small neuronal populations in dendrites encode for ‘related‘ memories (in time, space, or context) (Silva et al., 2009; Rogerson et al., 2014). Synaptic clusters, hence, may be considered as crucial computational and memory storage units in the brain.

### 3.1. Long term potentiation

Long-term potentiation (LTP) is viewed as the crucial trigger to consolidate synaptic connections and improve synaptic efficacy (Bliss and Lomo, 1973; Volianskis et al., 2015; Bliss et al., 2018). It is induced by rhythmic bursts of activity reminiscent of the theta rhythms typically occurring in hippocampus during learning (Grover et al., 2009). Properties of memory formation are critically dependent of the extent of LTP cooperativity, LTP consolidation, and of the ability for dendritic protein synthesis. Synaptic tagging depends on the availability of plasticity-related proteins (PRPs) that are either produced in the cell body or translated from pre-existing mRNAs in dendrites (Montarolo et al., 1986; Schacher et al., 1988; Scharf et al., 2002; Hernandez and Abel, 2008; Alberini and Kandel, 2014). Because synaptic growth at pre- and post-synaptic terminals depends on protein synthesis (Bailey and Chen, 1983, 1989), a delayed wave for the consolidation of long-term memory is required (Katche et al., 2010).

Specific mRNA expression in dendrites and protein synthesis induced in a synaptic spine could convert early-LTP of a nearby spine to late LTP via synaptic capture mechanisms as hypothesized in the synaptic tagging and capture (STC) model (Steward and Schuman, 2003; Cajigas et al., 2012).

An intriguing consequence of dendritic STC is that it can become a mechanism for associating temporally close memories, captured by nearby synapses. This mechanism could support the generation of functional and/or anatomical clusters of synapses facilitating cross-capture of proteins between synapses that express either LTP or LTD, and consolidating formation of memory engrams (Govindarajan et al., 2006).

### 3.2. Bifurcations, storage of information, and engram formation

**FIGURE 2:**
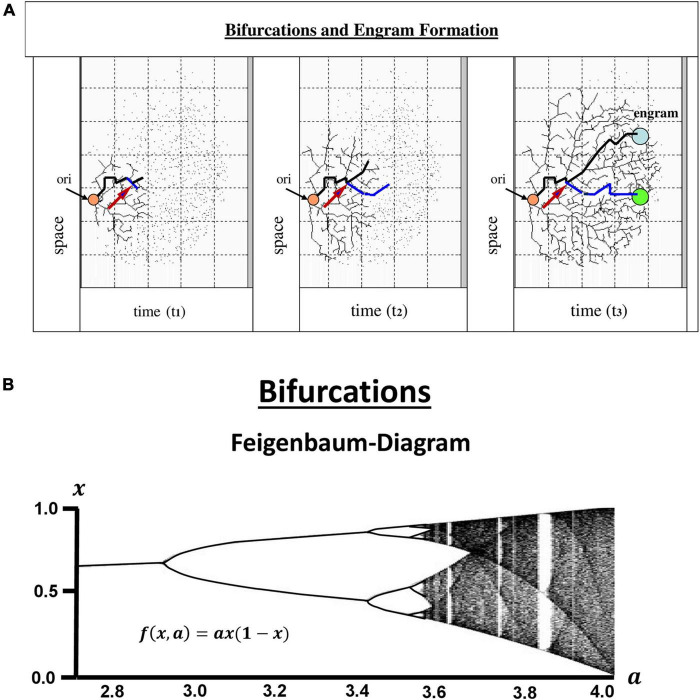
Bifurcations and engram formation. At some unknown point of origin (arrow ori) in one’s life there is a first decision-making between yes or no (0 vs. 1) followed by innumerable more bifurcations. This happens in each cell of the organism, but in human beings appears to be particularly interesting in the Central Nervous system. Obviously, those are events digital in nature, which raises the question of whether or no information processing and storage is comparable to computer devices **(A)**. The bifurcations exemplarily shown in the figure and their development over time display dynamic events reminiscent of the mathematical model of bifurcations, the Feigenbaum diagram **(B)**. It is constructed according to the differential equation in the inset. The diagram clearly shows, that after the second round of bifurcations the systems turns into a chaotic process with sporadic additional bifurcations embedded (where the Lyapunov exponent runs back to zero within the red line), but on the whole into a non-linear system almost completely devoid of digital events. In the brain, learning processes and memories stored in so-called “engrams” are founded on higher order information processing, storage and recall. Many of the bifurcations may have only little effects, but others may have strong impact during the whole life (a, arrow). There are several theories as to how the brain handles the wealth of information entering from the external world, either focusing on communication within neuronal networks and their oscillations, or putting more weight on the contribution of glial cells, on astrocytes in particular, and their information processing largely relying on analog events. Also, recently, engram cells have been identified in the hippocampus. But there is a high likelihood, that engrams are dispersed all over the brain, and to maintain the whole system, a higher order technology of hybrid computation is required. In contrast to computer technologies, however, the construction of the “hard disk” of memory engrams is time-dependent and irreversible. Nothing can be erased or reset to a previous time point to start again.

Beginning and development of human beings appear to be dependent on yes-no or either-or decisions comparable to the fundamental workings of electronic devices. Those bit-like events, or “bifurcations” may have little or larger consequences but altogether contribute to the development of an organism. A fundamental feature to all of them is their intrinsic “irreversibility.” There is no way to step back. The sum of bifurcations accumulating continuously in a human being is the result of a chaotic process, critically dependent on the time of onset and subsequently progressing during the whole life (Figure 2A), irreproducible in any other individuum, even in monozygotic twins, shaping personalities that are unique.

Bifurcations can be observed on all levels of an individuum, from organs to cells and to molecules. For those reasons, the question has been addressed many times, if the way a human brain works is comparable to a computer, working in binary modes. In mathematics, bifurcations have been intensely investigated since the seminal publications by Feigenbaum (1978, 1979). After a few steps of period doublings, the map dramatically changes into a chaotic appearance with some bifurcations embedded in the logistic map (Figure 2B). There is also a critical dependence on the initial conditions which is characteristic of non-linear systems. Moreover, the salient feature of the diagrams is their self-similarity, typical of chaotic systems, and highly reminiscent of fractals as described later by Mandelbrot (1980).

Are those fascinating results delivered by the most basic natural science equivalents of engrams formed in the CNS ?

Engrams are specific changes in the brain formed by experience (Semon, 1921) and stored in a quiescent state (Figure 2A) that becomes functional under conditions that lead to retrieval (Tulving, 1983) or in psychiatric disorders (Gebicke-Haerter, 2014). Although engrams have not been found in their entirety (Josselyn et al., 2017), significant progress has been made in engram research and theoretic models have been developed. According to Hebb’s (1949) influential theory, simultaneously activated synapses in clusters of neurons (e.g., by LTP) are reinforced, and this mechanism is the basis for learning and memory. Alternatively, newly established synaptic weights within an activated neuronal population may result in an engram. This would lead to an expanded storage capacity, because there are significantly greater numbers of combinations of synaptic weights than of neurons in any given cortical network. From these theories, one may conclude, that specific connectivity patterns between neurons are engrams (Redondo et al., 2014; Tonegawa et al., 2015b; Roy et al., 2017; Choi et al., 2018).

Alternative concepts are more in favor of the cellular aspect. And indeed, a number of studies have identified engram cells, distinct populations of neurons encoding engrams for specific memories (Han et al., 2007, 2009; Josselyn, 2010; Garner et al., 2012; Liu et al., 2012; Ramirez et al., 2013; Kim et al., 2014; Tonegawa et al., 2015a; Josselyn and Tonegawa, 2020), that appear to be distributed across multiple brain regions (Roy et al., 2022). These cells are conditioned by specific cues associated with incoming signals (Guzowski et al., 1999; Deng W. et al., 2013; Denny et al., 2014). Memory reactivation increased engram cell excitability, which enhanced retrieval of specific memory content (Pignatelli et al., 2019), and memory recall can be elicited by their stimulation (Ryan et al., 2015). For example, intrinsic excitability of dentate neurons results in self-assembly into a memory engram (Park et al., 2016). This has been shown in great detail by the Tonegawa lab, using hippocampus-dependent context fear conditioning (FC). Their data reveals interesting insights into false memory and valence reversal. Enhanced connectivity between CA3 to CA1 engram projections strongly disabled LTP. These events balancing excitation and inhibition have been termed homeostatic plasticity (Turrigiano and Nelson, 2004).

Molecular biology studies on the transcriptome of FC engram cells revealed genome-wide alterations during FC memory consolidation. In particular, the CREB network was activated exclusively in engram neurons (Rao-Ruiz et al., 2019). Amongst the top 50 differentially expressed genes, twenty-two were CREB-dependent genes including Arc, Atf3, Penk, Cdkn1a, Sorcs3, and Inhba. The upregulated genes Arc, Atf3, and Penk are involved in synaptic (Jancic et al., 2009) and structural plasticity (Pai et al., 2018). Apart from Arc (Link et al., 1995; Lyford et al., 1995; Nakayama et al., 2015), there are more genes as part of a “plasticity transcriptome” (plasticity-related genes) believed to be associated with long-term memory, such as Arcadlin (Yamagata et al., 1999), RB-3 (Beilharz et al., 1998), Syt4 (Vician et al., 1995), and Nrxn3, Adrb1, Grm6, Chrm4, Chrna4, Grin2D, Gad2 (Ryan et al., 2011). Expression of those genes induce and consolidate functional and structural long-term changes of neuronal connectivity following learning. Moreover, amongst differentially regulated ion channels, 11 were potassium channels. The voltage-gated K + channel Kcnq3 was 72-fold downregulated in engram neurons.

Molecular biology studies on long-term storage of memory (LTM) hypothesized an “intramolecular autocatalytic” reaction (Crick, 1984; Lisman, 1985; Roberson and Sweatt, 1999), a molecular mechanism that once activated persists in a self-sustaining manner. Protein-kinase-M-zeta (PKMζ), an atypical isoform of PKC, was a particularly interesting candidate to consolidate LTMs, because its mRNA is transported to dendrites and its translation is induced by LTP. PKMζ can be considered as a core molecular mechanism of late-LTP and maintenance of LTM, obeying the criteria of necessity, occlusion, erasure, and persistence. All known PKMζ inhibitors abolish this function, but they have no effect on early-LTP and basal synaptic transmission. An LTM trace can be associated with a discrete subset of neurons, reminiscent of engram cells. Those data stimulated studies on remote LTMs (i.e., a few weeks old or older), investigating the fate of memories during systems consolidation (for review see: Frankland and Bontempi, 2005). Systems consolidation progressively relies on cortical areas and less on the hippocampus in a process that involves delayed maturation of cortical neurons and may be mediated by hippocampal sharp-wave ripples (SWR). They are associated with highly synchronous neural firing of subsecond duration and support both memory consolidation and memory retrieval (for reviews see: Squire and Alvarez, 1995; Carr et al., 2011; Buzsaki, 2015; Foster, 2017; Joo and Frank, 2018; Tang and Jadhav, 2018; Tonegawa et al., 2018).

The extracellularly recorded sharp wave component of the SWR corresponds to the accumulated, synchronous depolarization of a large fraction of the neurons in the CA1 region of the hippocampus (Buzsaki, 1986). This effect may be induced by activities from CA3 neurons (Valero et al., 2017) which also excite interneurons. As a result, interneuron-coordinated pyramidal cell ensembles undergo oscillatory excitation and inhibition characterized as a high-amplitude (150–250 Hz), co-incident ripple (English et al., 2014; Stark et al., 2014). The distribution of ripple band power is approximately log-normal with a long tail toward high values, but not bimodal (Cheng and Frank, 2008). SWR rate is at its highest in the contexts of novelty and reward. Therefore, it likely serves to trigger subsequent, slower synaptic consolidation processes (Buzsaki, 1989). Hence, engram formation may be a two-step process.

An interesting understanding of modern engram theory is the view that consolidation depends on retrieval (Lisman et al., 2018). Retrieval is thought to occur if neural activity patterns in the hippocampus that correspond to those that occurred during a previous experience are reactivated. Retrieval appears to be occurring specifically in REM-phases of sleep, where dreaming is dominant and memories from various, seemingly random (engram) sources are surfacing unconsciously. Furthermore, retrieval of a single stimulus-response association can drive behavior directly or, confronted with multiple options, the brain may recall specific episodes of past experience for decision-making or planning, giving rise to new ideas. Retrieval may, hence, support imagination or intuition, which can be understood as the rearrangement or elaboration of stored information in the mental simulation of future possibilities (Josselyn and Frankland, 2018).

## 4. Epigenetics and information processing in long term memory

### 4.1. The epigenetic switchboard

Accumulating evidence supports the view that epigenetic mechanisms of gene regulation are critically involved in processes underlying learning and memory (Meadows et al., 2016; Sweatt, 2017).

At this point it is important to briefly refresh the biochemical events involved in transcription and translation in terms of digital and analog information processing.

**FIGURE 3:**
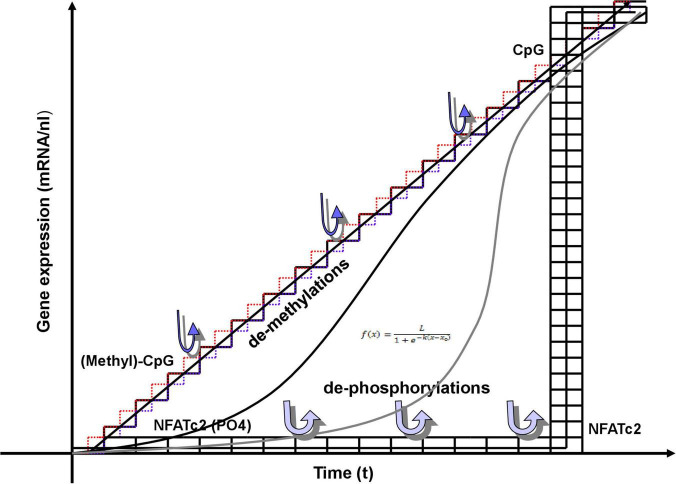
Digital and analog events involved in gene transcription. Epigenetic DNA and histone modifications, i.e., DNA methylations and posttranslational histone-tail modifications (PTT) are clearly digital. Demethylations, proceeding from methyl-CpGs at low transcription rates near origin result in increasing, step-wise transcriptions. They are shown as single steps along a straight line obeying the equation: *y* = nx. Infinitesimal approximations of the triangular (digital) demethylations could be adapted to the (analog) line of transcription. The combined effects of methylations and PTT fine-tune assembly of transcription initiation complex and subsequent transcription. Those effects may also result in logistic (sigmoidal) transcription rates described by (analog) non-linear differential equations, as shown in two more examples. The equation of logistic function or logistic curve (also known as sigmoid curve) entails a common “S” shaped curve defined by the equation in inset, where L = the maximum value of the curve; e = the natural logarithm base (or Euler’s number); x_0_ = the x-value of the sigmoid’s midpoint; and k = steepness of the curve or the logistic growth rate. Sigmoid curves are also very typical for enzyme reactions. The steepness is variable from very flat to very steep. Merging into a vertical line marks the transition into a digital behavior, as shown exemplarily with the transcription factor NFATc2. It is a kind of double-digital process. The protein is highly phosphorylated in its inactive (off) state, when residing in the cytoplasm. It is activated by stepwise dephosphorylation, that, however, do not show any visible effect (but probably increase the tension). Removal of the last phosphate results in overcoming a threshold to unleash its activity completely, entering the nucleus, binding to its DNA-binding site and inducing transcription.

Epigenetic control of gene expression begins with a relaxation of compact chromatin at sites of the genes to be activated. Those events are dependent on posttranslational modifications of histone proteins, and cytidine methylations or hydroxymethylations of DNA, all of which are clearly digital events. Cytosins in DNA can be (hydroxy-)methylated or not, and histones can be acetylated, methylated, phosphorylated, etc., or not. Neuronal activity can influence gene expression by dynamic DNA methylation (Figure 3; Nelson et al., 2008; Sharma et al., 2008; Guo et al., 2011; Halder et al., 2016). In excitatory neurons of the cerebral cortex, DNA methyltransferases (DNMTs), have been shown to modulate synaptic transmission (Levenson et al., 2006; Sweatt, 2016), synaptic scaling (Meadows et al., 2015), and neuronal excitability (Meadows et al., 2016). Conversely, de-regulated expression of DNMTs was associated to defects in the GABAergic system (Matrisciano et al., 2013) in patients with neuropsychiatric diseases like schizophrenia (Huang and Akbarian, 2007; Sananbenesi and Fischer, 2009; Gebicke-Haerter, 2012; Saradalekshmi et al., 2014; Benes, 2015), which strongly suggests important influences of DNMTs on inhibitory interneurons, as well.

The DNA-methylating activity of DNMT1 is often correlated with transcriptional repression (Bestor, 2000; Robertson K. D., 2002; Bordagaray et al., 2022). To investigate in detail how DNMT1 acts on GABAergic transmission, target genes have been studied in Dnmt1-deficient and WT interneurons by correlative global methylome and transcriptome analysis (Pensold et al., 2020). A significant number of differentially expressed genes were associated with clathrin-dependent endocytosis. Since the expression of numerous genes associated to the clathrin-mediated endocytosis pathway was upregulated and their methylation reduced upon Dnmt1 deletion, DNMT1-mediated DNA methylation likely exerts a direct regulation of endocytosis, slowing down vesicle recycling and ensuing presynaptic transmission.

Physiologically, ten–eleven translocation (TET) family enzyme-dependent mechanisms result in DNA demethylation of activity-regulated genes (Figure 3; Wu and Zhang, 2017; Wu et al., 2017) and subsequent memory extinction (Rudenko et al., 2013). TETs oxidize 5-methylcytosine (5mC) to 5-hydroxymethylcytosine (5hmC) that can then be actively reverted to cytosine. The regulation of synaptic transmission and surface levels of GluR1 receptors in hippocampal neurons has been shown to be mediated by TET3 DNA demethylation (Yu et al., 2015). Therefore, both demethylation and *de novo* DNA methylation are important for modulating neuronal plasticity and learning and memory in the adult nervous system (Lister et al., 2013; Sweatt, 2016). Basically, memory formation requires hypermethylation of memory suppressor genes and hypomethylation of memory promoting genes. One of those memory suppressor genes, calcineurin (CaN), showed increased methylation in cortical neurons up to 30 days after fear conditioning (Miller and Sweatt, 2007). The same is true for protein phosphatase 1 (PP1), while the synaptic plasticity gene reelin is demethylated and transcribed. At this point, it looks very likely that, within a certain time scale, adding switches of DNA methylation in some groups of genes and removing those switches from other clusters of specific genes creates new methylation patterns that pave the way for memory (engram) formation and consolidation.

### 4.2. Posttranslational histone modifications (PTM)

Proteins modifying histone tails are grouped into three categories; “writers,” “readers,” and “erasers.” “Writers” such as histone acetyltransferases (HATs) modify and prepare specific lysines in histones to be recognized by bromodomain (BRD) “readers” to bind to those acetylated lysines. BRDs were discovered as the first domain to exclusively bind acetylated lysine (Dhalluin et al., 1999). These PTMs are not permanent however, since “erasers” such as histone deacetylases (HDACs) are able to remove the acetylation PTM (Janzen et al., 2010). Since acetylated histones act as binding sites for the transcriptional machinery, histone acetylation is often associated with transcriptional activation. Due to the efficient activities of HAT and HDAC, histone acetylation is fast and reversible. Transcription and protein synthesis induced after learning are observed only during restricted periods of time, which means that there is a limited time frame for memory consolidation (Igaz et al., 2002). Histone phosphorylation may also induce transcription, while histone methylation can facilitate both transcriptional activation and repression (Levenson et al., 2004). Methylated histones are recognized by chromodomain containing plant homeodomain (PHD) fingers, discovered in 1993, known to bind histone H3 tri-methylated at lysine 4 (H3K4me3) (Aasland et al., 1995; Wysocka et al., 2006). Transcriptional activation or repression is dependent on the interaction of chromodomain-containing proteins with the specifically methylated lysine. Histone H3 di- and tri-methylation at lysine 9 (H3K9) results in transcriptional repression, while histone H3 methylation at lysine 4 (H3K4) is associated with transcriptional activation (Vermeulen et al., 2007). Similar to DNA methylations, the influence of histone methylations on gene expression are required for memory formation, as well. Compared to the above described patterns of DNA methylation, it is evident that the digital biochemistry of histone PTMs is orders of magnitude more complex and offers an unprecedented wealth of fine-tuning of storage and retrieval of memory.

### 4.3. Combined DNA methylation and histone PTMs and posttranscriptional events

Noradrenergic stabilization of heterosynaptic (“tagged“) LTP requires not only transcription, but specifically, DNA methylation and histone acetylation (Brandwein and Nguyen, 2019). During and after LTP-induced learning, the expression of a “maintenance transcriptome” has to be established and to remain active at least in the range of days. In this period of time, there appear negative epigenetic regulators of gene expression, particularly histone deacetylases, such as HDAC1 and 2, but also a variety of additional members of the HDAC family (Mahgoub and Monteggia, 2014; Penney and Tsai, 2014). Hence, the maintenance transcriptome negatively regulates the plasticity transcriptome, restraining the plastic capability of a neuron after learning. It elevates the threshold for changes in engram neurons and helps to stabilize new connectivites.

Furthermore, there are additional digital events during posttranscription, such as RNA editing and RNA degradation by miRNAs, controling the amount of RNA binding to ribosomes. The resultant quantities of those final mature RNAs can be grouped in more or less linear scales, i.e., again a digital-analog conversion. Finally, another digital-analog transition of biological information is associated with the specific aminoacylation of cognate tRNAs. The aminoacyl-tRNA synthetases (aaRS), on the one hand, specifically recognize individual amino acids, which after their activation are conjugated by aaRS to the cognate tRNA molecules (Ling et al., 2009). In this manner, the digital event of tRNA anticodon binding is translated into an analog string of information by adding amino acids and forming the three-dimensional structure of a protein. Here it is necessary to remember the basic principles and differences between the fundamental functions of DNA and proteins in biological systems in terms of digital and analog information processing (Koonin, 2015). We recall the Central Dogma of Sir Francis Crick (1970), saying that “there is no route of reverse information transfer from proteins to nucleic acids, i.e., no reverse translation.”

This is a fundamental difference between information processing and storage in computers and the Central Nervous System. Within the former, information can be completely erased. Or the system can be reset to any previous stage and can be started again from that point on. Corrections or replacements of entered and stored information are possible.

In the brain, there is an epigenetic switchboard of incomprehensibly large yes/no options that are adjusted in response to environmental impact and demands, and induce optimized adaptations during subsequent, additional digital events. Those mechanisms keep advancing in complex, non-linear ways determined by self-sustained switchboard reprofiling maintained during the whole life span of an organism. Although there is no way back, however, there are innumerable possibilities to correct existing and stored information, and to “endeavor” new possibilities. Admittedly, this is somehow reminiscent of unsupervised learning in computer systems. Nevertheless, it remains to be kept in mind that the unique, unidirectional flow of information transfer represents the shift from digital to analogous encoding of information. In other words, there is a transition between the fundamentally one-dimensional (digital) information contained in nucleic acids to the three-dimensional, analog form of information embodied in proteins (Haykin and Van Veen, 2003). This flow of information is unique to the brain and to biological systems in general.

The all-or-nothing modifications described above do not provoke yes-or-no transcription, but solicit graded transcription dependent on the combination and overall sum of all modifications allowing for successful assembly of the initiation complex. This may result in linear or more sigmoidal time-courses of gene expression (Figure 3). Hence, outcomes are analog events. However, there are also exceptions, where those modifications provoke all-or-nothing events.

For example, in Th2 lymphocytes the transcription factor NFATc2 is required for expression of IL-4. NFATc2 is phosphorylated in its inactive form outside the nucleus. It enters the nucleus for binding to the IL-4 promoter only, when it is completely dephosphorylated by the phosphatase calcineurin. Under these conditions, interleukin-4 is fully transcribed without running through any intermediate stages (Figure 3; Köck et al., 2014).

## 5. Additional computational dimension: astrocytes, and the tripartite synapse

For a long time information processing in brain has been attributed exclusively to neurons. However, accumulating data has assigned an even more important role to protoplasmic astrocytes and put forward the notion that they are instrumental in learning and behavior [reviewed by Wang and Bordey (2008), Verkhratsky et al. (2011), Parpura et al. (2012), Han et al. (2013), Volterra (2013)]. Apparently, they are not only necessary but also sufficient for new memory formation (Adamsky et al., 2018). The intimate embracement of synapses by thin astrocytic processes was coined the “tripartite synapse” (Araque et al., 1999; Perea et al., 2009). It postulates that the synapse can no longer be considered as only engaging two neuronal elements isolated from the rest of the parenchyma.

### 5.1. Interactions of astrocytes with synapses and neuronal circuits

However, not all synapses are in immediate contact with perisynaptic astrocytic processes (PAPs). They may engage and disengage from synapses spontaneously or in response to physiological (and pathological) stimuli (Panatier et al., 2006; Bellesi et al., 2015). During LTP induction, more PAPs become associated to activated synapses (Lushnikova et al., 2009; Perez-Alvarez et al., 2014), possibly supported by RNA translation within PAPs (Sakers et al., 2017). In neocortex, 30–60% of synapses are enwrapped by astrocytes (Reichenbach et al., 2010), 60–90% in hippocampus (Ventura and Harris, 1999; Witcher et al., 2007), and up to 90% in the somatosensory cortex layer IV (Bernardinelli et al., 2014). The numerous synaptic contacts assign an intriguing role to astrocytic processes in spreading signal information to groups of neighboring synapses, hence an involvement in heterosynaptic plasticity. This plasticity could extend to a number of dendrites even if they do not belong to the same neuron (so-called heteroneuronal plasticity), which could regulate switching between synaptic ensembles during information processing (Min et al., 2012). It is possible, therefore, that an individual astrocyte interferes with the function of all (or subsets of) synapses within its domain. On the other hand, synapses will be functionally divided in two contiguous segments governed independently from one another if a dendrite passes through the domains of two distinct astrocytes. This concept embodies an extra layer of complexity in our understanding of brain computation. Apart from the neuronal layout, polarity and connectivity, a mosaic of independent (though likely cooperating) astrocyte domains add additional control mechanisms to separate volumes of neuropil. Astrocytes affect spine maturation and the function of mature synapses in a “synaptic island”-restricted manner. Large neuronal dendrites may cross domains of hundreds of different astrocytes, which results in reprogramming various synaptic inputs by independent astroglial cells. Consequently, dendritic synaptic inputs not only are shaped by signals from multiple, incoming, pre-synaptic neurons, but also activities of multiple astrocytes embedding the dendritic network.

### 5.2. Astrocyte domains and the three-dimensional and seamless expression of consciousness and explicit memories

Ribonucleic acid expression is enhanced in neurons during excitation, and declines sharply afterward (De Robertis, 1964). After neuronal excitation, sustained increased RNA production has been observed in astrocytes, which coincides with the period of trace retention. This study made Luria to conclude that “the hypothesis that the glia is concerned in retention of memory traces is unquestionably one of the most important discoveries in modern neurophysiology and it must shed considerable light on the intimate mechanism of memory” (Luria, 1973).

Astrocytes are not electrically excitable, but they are well-known for both stimulus-induced and spontaneous intracellular calcium signals (Cornell-Bell et al., 1992). Those calcium signals usually do not propagate to neighboring astrocytes through gap junctions (Di Castro et al., 2011; Volterra et al., 2014), and the majority are observed in peripheral thin processes rather than in their soma. They do not result from mobilization of internal calcium stores (Srinivasan et al., 2015).

Communication between astroglia and neurons has profound impact on synaptic transmission. Astroglia contain neuronal excitability, release probability and insertion of postsynaptic AMPA receptors, which results in synapse silencing. This strongly impacts on the threshold balance between long-term potentiation and long-term depression (Pannasch et al., 2011). In the absence of functional astroglial networks (Cx30-/-Cx43-/- in hippocampal slices), postsynaptic activity was strongly amplified as a result of massive increase in synaptically-evoked firing (Wallraff et al., 2006).

### 5.3. Astrocytic fine-tuning of computation by gliotransmitters and transmitter receptors

Synaptic transmission can be significantly modified by specific proteins produced in astrocytic fibers (Heller and Rusakov, 2015), such as glutamate transporters (GLT1) (Chaudhry et al., 1995), glutamine synthetase (Derouiche and Frotscher, 1991), aquaporins (Thrane et al., 2011), potassium channels (Higashi et al., 2001), cell adhesion molecules (ephrin) (Zhuang et al., 2011), and lactate transporters (Puchades et al., 2013).

Furthermore, astrocytic release of (glio-) transmitters directly interacts with pre- or post-synaptic neuronal receptors stream-lining synaptic efficacy, potency or plasticity. For instance, astrocytic ATP, which is rapidly degraded to adenosine, may act on pre-synaptic neuronal A1R to inhibit pre-synaptic release (Schmitt et al., 2012) or on post-synaptic A2R receptors to potentiate synaptic strength (Gordon et al., 2005). Furthermore, stimulation of cholinergic muscarinic receptors in the somatosensory cortex (Takata et al., 2011) can be adjusted by the release of the NMDAR co-agonist D-serine (Rollenhagen et al., 2007; Papouin et al., 2012). This D-serine “boost” affects the threshold of NMDAR-activation, facilitating the receptor to trigger the downstream signaling pathway that underlies LTP induction (Papouin et al., 2017; Adamsky et al., 2018; Robin et al., 2018). Hence, transient release of D-serine by astrocytes at hippocampal CA1 synapses is necessary for NMDAR-dependent LTP (Yang et al., 2003; Panatier et al., 2006). This release affects LTP only at synapses located within the domain of this astrocyte and not LTP at synapses located in the domain of a neighboring control astrocyte (Henneberger et al., 2010). Astrocytic D-serine also mediates integration of adult-born granule neurons into the hippocampal circuitry (Sultan et al., 2015), a process that is ongoing throughout life and may alter local circuit performance in memory processes and mood control (Toni and Schinder, 2015). The D-serine-controlled synaptic NMDAR impact on sleep–wake cycle clearly relies on analog computation, associating vigilance state to memory formation. During wakefulness, a steady accumulation of sleep-promoting substances enhance the pressure to sleep. Those substances are then gradually degraded. Sleep–wake cycles in rodents have been shown to undergo neuronal network oscillations sustained by astrocyte-derived adenosine. Slow-wave oscillations (<1 Hz), in particular, observed during non-rapid eye movement (NREM) sleep have been associated with memory consolidation (Marshall et al., 2006; Halassa et al., 2009).

Glutamate released by astrocytes into the synaptic cleft modifies axonal conduction, broadens action potentials (Sasaki et al., 2011), and can transiently enhance presynaptic transmitter release (Jourdain et al., 2007; Perea and Araque, 2007; Navarrete and Araque, 2010). Moreover, astrocytic glutamate also targets neuronal dendrites as shown with recordings from hippocampal CA1 pyramidal neurons. Resulting dendritic plateau potentials (Ashhad and Narayanan, 2016) have been implicated in localized plasticity and spatial memory formation (Bittner et al., 2015).

Furthermore, astrocytic l-lactate plays a key role in LTP at hippocampal CA1 synapses. It is stored as glycogen in astrocytes, metabolized to l-lactate during periods of high energy demand, and shuttled to neurons (Pellerin and Magistretti, 1994). LTP in CA1 and CA3 was blocked *in vivo* when its production was inhibited in astrocytes, suggesting an important role for l-lactate in long-term episodic memory (Suzuki et al., 2011).

Astrocytes express virtually all neurotransmitter and neuromodulator receptors (glutamate, dopamine, norepinephrine, acetylcholine, serotonin, and GABA) (Kettenmann and Zorec, 2013). Individual astrocytes may co-express as many as six different receptors (Shao et al., 1994). But their expression may be region-specific in that, for instance, dopamine receptors are found in astrocytes of the substantia nigra (Miyazaki et al., 2004), and in prefrontal cortex (Khan et al., 2001), whereas glutamate receptors are encountered throughout gray matter witnessing the wide-spread release of glutamate by excitatory synapses everywhere in the CNS. Due to this occurrence, this transmitter is the best candidate to be involved in consciousness and memory formation provided that consciousness and memory are disseminated all over the brain (Calvin, 1996; Cooper et al., 2003; Jones, 2005; Posner et al., 2007). Moreover, adrenergic receptors are more abundant in astrocytes than in neurons (Stone and John, 1991; Aoki, 1992). Although ß-receptors expressed by hippocampal neurons were viewed to potentiate LTP and memory, more recent studies revealed that astrocytic β-2-adrenoceptors are more important, because the known positive effect of arousal on memory performance could be associated to the finding that a key part of the noradrenergic effect is mediated by astrocytes. Moreover, acute stress triggers noradrenaline release activating astrocytic β-2-adrenoceptors, which may increase cognitive performance. Conversely, prolonged stress with sustained astrocyte activation impaired cognitive performance. This has been shown by administration of a β-2 agonist over days, improving memory performance, whereas more extensive exposure to the drug resulted in decline of cognitive ability (Dong et al., 2017). O’Donnell et al. (2012) emphasize that “norepinephrine signaling to astrocytes is necessary to drive the transformation of memory from short to long-term stores” and “is important for supporting processes that bridge short to long-term behavioral adaptation.” Obviously, all those events do not obey an all-or-nothing regimen, as realized in computer memory devices.

Acetylcholine, which is released during vigilance states by long range neuronal fibers, also activates astrocyte acetylcholine receptors and promotes astrocyte-mediated neuronal cross-talk (Araque et al., 2002; Perea and Araque, 2005; Navarrete et al., 2012; Papouin et al., 2017). Acetylcholine in concert with noradrenaline maintain brain-wide oscillations to synchronize different brain areas and to insure correct cognitive performance and sensory perception (Wang, 2010).

**FIGURE 4:**
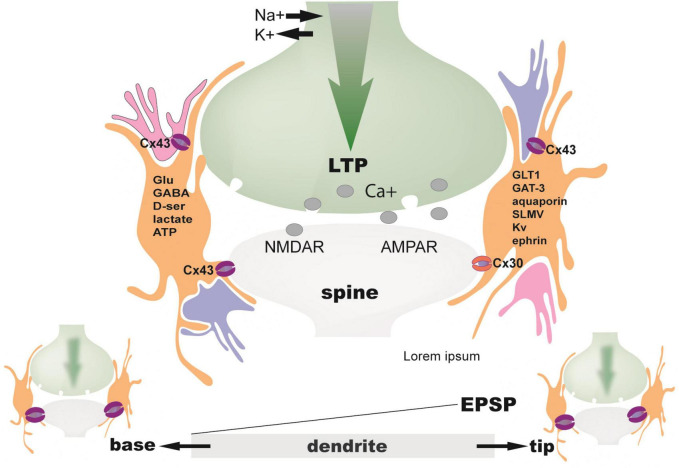
The tripartite synapse. Ensheathment of synaptic spines by perisynaptic astrocytic processes (PAPs) can change over time. It depends on neuronal activity and ensuing actin-dependent motility in PAPs. At high neuronal activity (LTP), activated synapses become ensheathed by more PAPs. One astrocyte may contact 300–600 dendrites and up to 36 spines per dendrite (Halassa et al., 2007). Those dendritic segments with their synaptic spines are under strict control of processes from only this astrocyte delineating its territory: orange (Bushong et al., 2002). That means that an individual astrocyte handles a defined volume of neuropil. There is no interference with other astrocytes. Only this astrocyte is responsible for surveillance and control of neuronal elements within this domain. Therefore, a single astrocyte theoretically oversees in its territory 20,000–160,000 individual synapses in the rodent brain and approximately 270,000 to 2 million synapses in the human brain (Oberheim et al., 2009; Heller and Rusakov, 2015). Because, however, an individual astrocyte affects the function of synapses solely located within its domain, a dendrite passing through the territories of two distinct astrocytes will be functionally divided in two contiguous segments governed independently from one another, as far as synapses are concerned. Decisions are made in dendrites far more often than in the cell body, which underscores the complex and highly non-linear capabilities of information processing within the dendritic tree. Such computations are not just digital, but also analog. For example, dendritic spikings are not stereotypic events. Amplitudes of EPSPs and the supralinearity of electrical integration during the stimulation of multiple synapses, e.g., by LTP, vary from the base to the tip of a single dendrite. For example, the base or the middle section of the dendrite show lower EPSP supralinearity, lower EPSP amplitude, and lower gain compared to the tip (Branco and Häusser, 2011). Moreover, the positioning of excitation along the dendrite is crucial for the amplitude and threshold of basal dendritic spikes (Behabadi et al., 2012). Proximal excitation lowers the threshold for spike generation and increases the voltage gain of distal inputs, whereas distal excitation lowers the threshold for dendritic spike generation in more proximal inputs. Spiking, then can be transmitted to astrocytes via gap junction channels (Cx43) and buffered as bits of information in the astrocytic syncytium. Memory, therefore, reminiscent of structures in electronic devices, appears to be stored both in form of RAM on the neuron level and in hard discs of astroglial networks. Apart from the involvement of astrocytes in analog information processing, there is also neuronal dendro-dendritic gap junction communication, adding another level of complexity in computation. Specific products made and released by astrocytes at synaptic spines have considerable influences on processing of arriving neuronal signals. Astrocytes release neurotransmitters (gliotransmitters), cotransmitters, like D-serine, or ATP, converted into adenosine, and express respective neurotransmitter receptors and glutamate transporters (GLT1) (Chaudhry et al., 1995), glutamine synthetase (Derouiche and Frotscher, 1991), aquaporins (Thrane et al., 2011), potassium channels (Higashi et al., 2001), cell adhesion molecules (ephrin) (Zhuang et al., 2011), and lactate transporters (Puchades et al., 2013). Astrocytes can also communicate via exocytosis of synaptic-like microvesicles (SLMV) (Vardjan et al., 2019).

Furthermore, stimulation of astrocytic endocannabinoid receptors (CB1Rs) at layer L4–L2/3 cortical synapses is required to induce spike-timing-dependent long-term depression (LTD) via activation of presynaptic NMDARs (Min and Nevian, 2012). Moreover, astrocyte CB1Rs are necessary to induce the classical NMDAR-dependent LTP at CA1 hippocampal synapses (along with astrocyte D-serine) (Robin et al., 2018; Figure 4).

In summary, along with detection of neurotransmitter by the postsynaptic neuron, astrocytes detect small amounts of neurotransmitter released presynaptically. They sense the level of neuronal activity at any given time (Pasti et al., 1997; Panatier et al., 2011) and integrate information conveyed at each synapse (Fellin and Carmignoto, 2004; Perea and Araque, 2006; Araque, 2008; Volterra, 2013). Therefore, synaptic information is simultaneously secured in a dynamic global matrix of innumerable astrocyte domains (Fellin, 2009; Parpura et al., 2012).

Tewari et al. (2016) report that astrocytes can: (1) facilitate or depress synaptic plasticity (De Pittà et al., 2016), (2) synchronize CA1 neuronal firing (Fellin et al., 2004), (3) modulate extracellular field potentials (Lee et al., 2014), (4) repair damaged synapses (Wade et al., 2012), and/or (5) initiate epileptic discharges (Reato et al., 2012; Tewari and Parpura, 2013).

### 5.4. Computational role of astrocytic calcium

It has been shown *in vitro*, *in situ*, and *in vivo* that [Ca2 + ] I release by astrocytic occurs as rapidly as in neurons (within 500 ms or less) (Winship et al., 2007; Marchaland et al., 2008; Chuquet et al., 2010; Santello et al., 2011). Therefore, astrocytic rapid responses are “compatible with a physiological role in fast activity-dependent synaptic modulation” (Santello et al., 2012; Kastanenka et al., 2020). This communication with neurons is ensured by expression of virtually all types of ionotropic receptors (Lalo et al., 2011; Steinhauser et al., 2013). Astrocyte synaptic-like currents have been shown to be triggered by neuronal activity *in vitro* and *in situ* (Dani et al., 1992; Porter and McCarthy, 1997; Matthias et al., 2003; Bergles and Edwards, 2008).

Conversely, rapid rises and long-lasting Ca2 + transients can be evoked in astrocytic perisynaptic processes, several micrometers long and in 3-dimensional space, by a single action potential (Di Castro et al., 2011; Panatier et al., 2011). Those Ca2 + -currents, which may last for seconds, support a role for astrocytes in working memory (Han et al., 2012). Studies of cholinergic (Takata et al., 2011) and noradrenergic neuromodulation (Ding et al., 2013; Paukert et al., 2014) revealed additional, slowly increasing somatic Ca2 + transients in the range of tens of seconds. In hippocampus, those Ca2 + transients can induce long-term effects on synaptic connections associated with memory formation (Adamsky et al., 2018).

It has to be mentioned that the notion of Ca2 + -dependent gliotransmission, the role of astrocytes in long-term potentiation (LTP), and whether D-serine is a gliotransmitter have been discussed, as reviewed in Bazargani and Attwell (2016) and Savtchouk and Volterra (2018). However, it has been well studied that, unlike in other glia, induction of metabotropic calcium waves in astrocytes coincides with electrical currents of synaptic activity in neighboring neurons (Murphy et al., 1993). Those electrical currents could spread via gap junctions and enable long-range astrocyte-neuronal synchrony (Szatkowski et al., 1990). Astrocytes reportedly form extensive networks of electrically coupled cells (Dermietzel et al., 1989). This network communication modulates pre-to-postsynaptic signaling by fine-tuning amplification of neuronal activity. Electrical coupling of astroglia forms an important part of intercellular communication between neuronal and tripartite synaptic activity. In terms of computation, those are interesting examples of a one-hit impact triggering a variety of subsequent, long-term analog processes. Crucial elements involved in this communication are gap junctions.

### 5.5. Astrocytic gap junctional computing

The most abundant connexin in the brain is the astrocyte-specific Cx43. In contrast to Cx32 and Cx26, Cx43 forms permeable channels. Mice lacking Cx43 (Cx30-/-Cx43-/- mice) showed amplified and extended fEPSP supposedly due to the combination of: (1) enhanced and longer-lasting extracellular potassium levels, and (2) accumulation of extracellular glutamate due to impaired astroglial clearance rate. Hence, precise neuronal communication depends on intact astroglial gap junctional networks, because they provide the large uptake capacities and fast redistributions of extracellular potassium and glutamate via astrocytic networks (Pannasch et al., 2011). Mice lacking connexin-30 show enhanced astrocytic glutamate uptake, diminished LTP expression, and repressed fear memory (Pannasch et al., 2014). In the same way, astrocytic glutamate uptake was increased and hippocampal LTP was reduced in mice deprived of the neuronal ephrin A4 receptor or its astrocytic ligand, ephrin A3 (Filosa et al., 2009), and dendritic spine morphology was altered (Murai et al., 2003).

Furthermore, the notion of a “generalized functional astrocytic syncytium” received strong support by the observation of intercellular calcium waves spreading to numerous cells by traveling through gap junctions (Mugnaini, 1986). Those decisive discoveries lent strong support to the idea that the syncytium embodies the basic structure of memory storage in the brain (hard disc), strongly reinforcing Galambos’ original assertion (Galambos, 1961). Gap junction coupling within this syncytium fulfils a neuroprotective role in that it is able to maintain a physiological membrane potential in the presence of elevated extracellular Kþ concentration and moreover can efficiently distribute excess Kþ across the syncytium. This helps to delay or inhibit the induction of spreading depolarizations. Apart from involvement of gap junctions in potassium buffering, also activity-dependent Na + spreads can transmit ionic currents through gap junction networks (Langer et al., 2012). All those ionic movements can be classified as analog computational events.

Astrocyte microdomains, which are quasicrystalline gap junctional plaques, approximately 1.5–12 um in diameter, are considered as the basic structures of postsynaptic information processing. Those plaques are believed to become assembled into packages of memories by crystallization into a long-lived highly resistant state and may be activated during consciousness (Robertson J. M., 2002). Indeed, an ultrastructural study reports that “interastrocytic gap junctions are packed in a crystalline array” (Massa and Mugnaini, 1982).

Additionally, astrocytes express heterotypic gap junctions that specifically connect to and communicate with all other macroglia and vascular elements forming a functional “panglial syncytium” (Nagy et al., 2003; Theis and Giaume, 2012). This integrative system of glial communication leads Fields to conclude that “glial cells are engaged in a global communication network that literally coordinates all types of information in the brain” and that “such oversight and regulation must be critical to brain function, and neurons are incapable of it” (Fields, 2009).

Moreover, it has been shown that siRNA can use gap junctions to travel from one cell to another and modify gene expression in the recipient cell (Valiunas et al., 2005). In this way, the astroglial syncytium is fundamental for the formation of long-term memories by epigenetic regulation of DNA throughout the brain.

This syncytium is currently viewed as a complex heterogeneous system that is multifunctional and closely regulated (Giaume et al., 2010; Hervé et al., 2012). It is centrally located between individual synapses and global neuronal networks (Robertson J. M., 2002). Astrocytes modulate both [reviewed by Halassa and Haydon (2010), Verkhratsky and Parpura (2013), Volterra (2013)]. Therefore, it has been put forward, that the astroglial syncytium is the primary coordinator of brain information processing, including consciousness (Pereira, 2007; Pereira and Furlan, 2010; Mitterauer, 2013), memories (Caudle, 2006; Banaclocha, 2007), intentionality (Mitterauer, 2007), and development of motor responses (Hassanpoor et al., 2012). Additionally, the glial network has been proposed as the “true substrate for information processing”–“where the thoughts dwell” (Verkhratsky and Toescu, 2006), synonymous with the “mind,” and the manifestation of the “global workspace” (Pereira and Furlan, 2009). Such a critical position suggests that this massive structure of interconnected astrocyte domains forms the body of the computational power of the brain.

### 5.6. Theoretical concepts

Any adverse effect on the computational tasks of astrocytes delineated above could significantly interfere with neuronal computation. Neurons distinguish incoming stimuli within a few milliseconds as individual entities, whereas astrocyte Ca2 + transients, the tentative astrocytic substrates of neural computing, are too slow to encode ultrafast representations (Vardjan et al., 2016). Obviously, this property serves as a complementary manner to cover various time scales. As stated by Murray, “the brain characteristically operates in parallel on a gradient of time scales that are nested and hierarchically organized” (Murray et al., 2014). For instance, attention and decision making, as well as the surge of emotions may take seconds, mood may change in minutes. Time scales of circadian rhythms are in the range of hours, and other life events with impact on learning and memory may extend to even longer time scales in the range of weeks, or years (Hari and Parkkonen, 2015).

Computationally, attention consists of a gain change (in amplitude of response or contrast) that results in the prioritization of relevant inputs over irrelevant information (Thiele and Bellgrove, 2018). Astrocytes could assist to identify signal coincidence and help prioritize information by regulation of gain. Variations of Ca2 + -dependent glutamate uptake may impede or enhance excitatory synaptic drive (Schummers et al., 2008) or excitatory and inhibitory neurotransmission (Perea et al., 2014). Regulation of gain may also encompass gliotransmission (Takata et al., 2011) and intrinsic neuronal excitability (Sasaki et al., 2012). Regulation of excitatory synaptic strength through gain control can be achieved by lowering glutamate uptake (Poskanzer and Yuste, 2016), by enhancing glutamate release (Halassa et al., 2009), or by GABA-uptake via GAT-3 transporters (Shigetomi et al., 2011).

The involvement of astrocytes in cortical slow oscillations (<1 Hz) (Poskanzer and Yuste, 2016) underlines the involvement of astrocytes in network activity beyond tripartite synapses. Slow oscillations are believed to be the default mode of cortical network activity (Sanchez-Vives et al., 2017). In this light, the notion has been put forward that neurons transmit instructions to astrocytes to make other neurons modify their activity via canonical computations.

Hence, neurons may imprint external signals like odors, position, images, words, abstract categories, and executive functions on networks, but astrocytes enable them to design and to operate canonical computations in local mini-circuits within larger-scale networks. One may hypothesize that those canonical computations are manifestations of computation of error-related statistics and/or time in different contexts.

Astrocyte-mediated filtering of synaptic transmission (denoted as “astrocyte-like control”) involves formation of so-called logic gates. Logic gates are essential building blocks in neural circuits to perform logic Boolean operations such as AND, OR, NOT, XOR, and NAND (Binder et al., 2007). Simple combinations of astrocytes and synapses comparable to the abovementioned mini-circuits might, in principle, allow for computation of any real-world function in a scalable manner (Song et al., 2016).

Therefore, neuron-focused studies should be viewed as computational elements within astrocyte mini-circuits, because dendrites and spines are embedded in an astrocyte “matrix” (Robertson, 2013). Since astrocytes participate in neuromodulation (Ding et al., 2013; Paukert et al., 2014), they might encode precision by temporally compensate prediction errors resulting from multiple synapses in astrocyte mini-circuits, to warrant sufficient statistics. The variable “precision” or “standard error” may be improved within a range of seconds by neuromodulators. Those molecules produce slower and more diffuse effects than transmitters, which eventually results in generation of brain states. State-dependent excitability of neuronal networks is associated with specific cognitive functions (Friston, 2009; Stephan et al., 2015).

During induction of synaptic plasticity, slow temporal properties of astrocytes could be essential to maintain the history of past activity (Min and Nevian, 2012). Indeed, computational models predict, that astrocytes improve synchronization of firing, and synaptic coordination (Amiri et al., 2013). Networks are tuned to oscillatory rhythms underlying memory processing (Tewari and Parpura, 2013), and integration of astrocytes improves network performance (Porto-Pazos et al., 2011; Fields et al., 2014). Within the syncytium, astrocytes may coordinate the excitability of functional neuronal ensembles and support their energetic demands (Chever et al., 2016; Clasadonte et al., 2017).

It looks as if at those levels analog information processing prevails, which leads to the conclusion, that even at relatively high levels of precision in the cell, analog computation is more efficient in its use of resources than deterministic digital computation.

## 6. Concluding remarks

Here we would like to reiterate to the central issue of this endeavor: Is The Human Brain Analog Or Digital?

This question stems from the knowledge of modern computer technology as described at the beginning of this review. The fundamental difference, however, is that the brain makes use of biomolecules for computation. All interactions of those molecules are distinguished by a probabilistic, analog nature. Because information is based on statistical approximations, the brain is non-deterministic and not “digital” (Sarpeshkar, 2010, 2014). On the other hand, many signals sent around the brain use “either-or” states. An action potential is triggered, a cytosine is methylated or not. These events are fundamental elements of communication in brain, as well. However, the binary arithmetic, binary logic or binary addressable memory of a computer chip are in no way sufficient to entail the full computational power of a neuron. The inevitable noise is attenuated by computation relying on feedback loops. Moreover, this type of computation not only involves neuronal networks and their oscillatory behavior, but also (astro-)glia networks mutually and intimately connected, which encompasses higher order information processing and more sophisticated ways of storing, consolidating, and retrieving memories than in hard discs of computers.

Along those lines, molecular parts of neural cells like ion channels, receptors, or enzymes as units of information processing simply cannot be understood as elements of digital, analog nor even hybrid computation. Supervision and control is embedded in various levels of cellular and molecular communication representing a system of more than sufficient flexibility to react and adapt to environmental challenges. Every single cell in the CNS can be viewed as a specific mini computer endowed with all the necessary tools to process incoming messages adequately along with efficient means to communicate with others in cellular and molecular networks. It is endowed with many molecular nanomachines executing their tasks inserted in the plasma membrane, cytoplasma, or in the nucleus almost frictionless and with close to 100% efficiency. A fascinating example of an analog-digital hybrid machine is the F0/F1-ATPase (Abrahams et al., 1994) located in the mitochondrial membrane, that phosphorylates ADP during clockwise rotation of its shaft (F0) injecting approx. 80 pN nm (close to the free energy of ATP) and dephosphorylates ATP turning counterclockwise (F1). The shaft’s driving force is provided by hydrogen current (“a proton-driven motor”) (Kinosita et al., 2000), which can increase or slow down the propelling speed and resultant production of nucleoside/nucleotide, controlling the production on demand. Another example is the kinesin/dynein system mediating fast axonal (anterograde/retrograde) transport of organelles on microtubules (Vale, 1987). Scrutinizing the literature in this respect easily reveals abundant similar examples of higher order computation everywhere in the Central Nervous System.

In conclusion, it has to be acknowledged that the brain entails many more computing options than any supercomputer. It has been programmed by nature and not by human beings. It is hard to imagine that a man-made computer program will be able to perform complex, abstract tasks like anticipation, intuition, or express social behaviors as basic requirements to live within human populations. All of those need adquisition, reinforcement and long-term consolidation. And, last not least, unlike in electronic devices, there is no option to “erase a folder” or to reset the whole system to a certain, previous condition. There is still a lot to learn and to understand about the computational power in our brain assembled and combined during tens of thousands of years by Nature. It is a big challenge but fascinating.

## Author contributions

The author wrote and revised the text and constructed the figures.
